# Release of copper-amended particles from micronized copper-pressure-treated wood during mechanical abrasion

**DOI:** 10.1186/s12951-016-0232-7

**Published:** 2016-11-28

**Authors:** Chiara Civardi, Lukas Schlagenhauf, Jean-Pierre Kaiser, Cordula Hirsch, Claudio Mucchino, Adrian Wichser, Peter Wick, Francis W. M. R. Schwarze

**Affiliations:** 1 Laboratory for Applied Wood Materials, Empa, Lerchenfeldstrasse 5, 9014 St. Gallen, Switzerland; 2 Institute for Building Materials, ETH, Stefano-Franscini-Platz 3, 8093 Zurich, Switzerland; 3 Empa, Functional Polymers, Dübendorf, Switzerland; 4 Empa, Analytical Chemistry, Dübendorf, Switzerland; 5 Institute for Environmental Engineering, ETH, Zurich, Switzerland; 6 Particles-Biology Interactions, Empa, St. Gallen, Switzerland; 7 Dipartimento di Chimica, Università degli Studi di Parma, Parma, Italy

**Keywords:** Cytotoxicity, Copper particles, Debris, Exposure, Inhalation, Wood dust

## Abstract

**Background:**

We investigated the particles released due to abrasion of wood surfaces pressure-treated with micronized copper azole (MCA) wood preservative and we gathered preliminary data on its in vitro cytotoxicity for lung cells. The data were compared with particles released after abrasion of untreated, water (0% MCA)-pressure-treated, chromated copper (CC)-pressure-treated wood, and varnished wood. Size, morphology, and composition of the released particles were analyzed.

**Results:**

Our results indicate that the abrasion of MCA-pressure-treated wood does not cause an additional release of nanoparticles from the unreacted copper (Cu) carbonate nanoparticles from of the MCA formulation. However, a small amount of released Cu was detected in the nanosized fraction of wood dust, which could penetrate the deep lungs. The acute cytotoxicity studies were performed on a human lung epithelial cell line and human macrophages derived from a monocytic cell line. These cell types are likely to encounter the released wood particles after inhalation.

**Conclusions:**

Our findings indicate that under the experimental conditions chosen, MCA does not pose a specific additional nano-risk, i.e. there is no additional release of nanoparticles and no specific nano-toxicity for lung epithelial cells and macrophages.

**Electronic supplementary material:**

The online version of this article (doi:10.1186/s12951-016-0232-7) contains supplementary material, which is available to authorized users.

## Background

Thousands of tons of wood chips and sawdust are being generated each day by industry, domestic environment, or improper disposal of debris. Further, the presence of wood preservatives may pose an environmental and human health risk due to release of toxic metals like arsenic and copper (Cu). Such an exposure pathway has already been recognized for various preservatives, in particular for chromated Cu arsenate (CCA) [1, 2]. We are currently experiencing an increased use of particulate Cu wood preservatives in order to effectively protect wood from decay and lengthen its service life. More specifically, basic Cu carbonate particulate systems with a size range between 1 nm and 25 μm were introduced for wood protection in the US market in 2006 [3]. This has resulted in more than 11,800,000 m^3^ of wood treated with micronized Cu (MC) formulations [4], which corresponds to over 75% of residential lumbers produced in the US [4].

Micronized Cu wood preservatives include a nanosized fraction of basic Cu carbonate, which may be of high concern: there is a strong indication that different Cu-based nanoparticles (NPs) have a high toxicity for aquatic organisms [5–10], terrestrial plants [11], mammals [12–17], and humans [18–23].

To date, the environmental fate of Cu carbonate particles from MC-pressure-treated wood has mostly assessed their leachability [24–28]. However particles generated by abrasion of MC-treated wood may be more hazardous than wood dust untreated or treated with conventional wood preservatives, due to the presence of Cu-based NPs. Platten et al. [29] and Santiago-Rodríguez et al. [30] recently assessed how exposure to Cu from wood dust originated from MC-pressure-treated wood can occur via dermal transfer or oral ingestion. Therefore, it is extremely important to determine the dust composition that can be inhaled after exposure—occupational or not—to abraded particles from MC-pressure-treated wood and its hazard to human lungs.

The current study characterizes the particles released from MC azole (MCA)-pressure-treated wood and compares them with particles generated from wood untreated, pressure-treated with the conventional wood preservative chromated Cu (CC), and with varnished untreated and MCA-pressure-treated wood. Subsequently, it assesses acute cytotoxic reactions of MCA, its components tebuconazole and Cu^2+^, as well as particles abraded from MCA-, CC-pressure treated wood and untreated wood to lung epithelial cells and macrophages.

## Methods

### MC characterization

We used a commercially available MC azole (MCA) formulation. This is the same as the formulation with high amount of tebuconazole MCA_HTBA we used in a previous investigation [31]. A full characterization of the Cu particles in the MCA formulation is available from the latter study. To summarize briefly, the measured particle size distribution of MCA was 104 ± 1.7 nm with an average zeta potential of −21 ± 0.4 mV.

### Wood sample preparation

Octagonal specimens of Scots pine (*Pinus sylvestris* L.) sapwood (90 mm diameter × 20 mm height) were used for the abrasion study. The specimens were prepared and pressure-treated with 2% aqueous suspensions of MCA or CC reference preservative, prepared according to the European standard ENV 807 [32]. After an 8-week drying procedure, some of the MCA-pressure-treated samples were coated three times with intervals of 24 h with a primer, i.e. solution of deck lacquer (90%) and white spirit (10%). The control materials were composed of: untreated wood samples and samples pressure-treated with a 0% MCA solution in distilled water, varnished untreated wood samples.

### Abrasion setup

The experimental setup has been described by Schlagenhauf et al. [33]. To simulate the abrasive process, a Taber Abraser (Model 5135, Taber, North Tonawanda, NY) was used. While the wood sample rotates, the Taber Abraser uses one abrasive wheel that abrades the sample continuously at the point of contact. The sample rotates 60 times/min and the weight applied on the wheel is 0.75 kg. The samples were abraded with S-42 sandpaper strips (Taber) mounted on a CS-0 (Taber) rubber wheel. A conductive silicone tube (TSI) with a rectangular inlet at the tube entrance with a 4.8 mm^2^ suction area was placed directly behind the abrasion area to collect the particles. The air flow was driven by a pump (N816.1.2KN.18, KNF, Germany). Devices for aerosol characterization and particle collection were included in the tubing system.

### Wood dust characterization

The generated particles were characterized in triplicates both in the aerosol form by particle size distribution measurements with an aerodynamic particle sizer (APS, Model 3321, TSI, Shoreview, MN) and a scanning mobility particle sizer (SMPS) consisting of a differential mobility analyzer (DMA) equipped with a long DMA column (Model 3080, TSI) and a condensation particle counter (CPC) (Model 3775, TSI). During each measurement, three particle size distributions were recorded. The recording time for each distribution was 195 s. The background distribution (without abrasive processes) of each experiment was measured three times. The experimental setup was verified by means of an atomizer aerosol generator (Model 3079, TSI). The particle size distributions obtained were processed as described by Schlagenhauf et al. [33]. In addition, the particles were collected on stubs and analyzed by means of scanning electron microscopy (SEM, Hitachi S-4800; Hitachi High-Technologies US and Canada, Illinois, USA). The stubs were plasma gold-sputtered (Polaron Equipment, SEM coating Unit E5100, Kontron AG, Switzerland; 5 mA, 1 mbar) prior to image acquisition.

The presence of Cu in the generated particles was assessed in the collected particles through ICP-MS (PerkinElmer Elan 6100, detection limit: 0.004 µg/L) and two distinct ICP-OES (Perkin-Elmer OPTIMA 3000, Jobin–Yvon HORIBA Ultima 2, detection limit for both instruments: 0.005 mg/L) instruments. In this way, we could benefit from the two different detection limits, as well as identify any effect of the instrumentation and–especially–of sample preparation on the detected amount of Cu. Analyses were carried out on the whole size range of abraded particles and on particles <1 μm collected on Nucleopore track-etch membrane filter (111106, pore size 0.2 μm, Whatman, UK). For ICP-MS and Perkin-Elmer OPTIMA 3000 ICP-OES Cu content analysis, the collected particles were dissolved nitric acid (HNO_3_, 65%, Supra Pure) and hydrogen peroxide (H_2_O_2_, 30%, Supra Pure) and subsequently underwent microwave digestion (MLS 1200 MEGA, Milestone, Leutkirch, Germany). Cu plasma standard solutions (1 g/L) were used for calibration. For Jobin–Yvon HORIBA Ultima 2 ICP-OES analysis a similar procedure was used, but without the addition of hydrogen peroxide. The detector voltage was set using a 100 mg/L standard solution, while a 7 levels calibration curve was employed for quantification.

### Cell culture

The human alveolar epithelial cell line A549 (ATCC: CCL-185) was grown in Roswell Park Memorial Institute (RPMI-1640) medium (Sigma-Aldrich) supplemented with 10% fetal calf serum (FCS) (Lonza), 2 mM l-glutamine (Gibco), 50 µg/mL penicillin (Gibco), 50 µg/mL streptomycin (Gibco), and 100 µg/mL neomycin (Gibco) at 37 °C in a humidified atmosphere containing 5% carbon dioxide (CO_2_, hereafter referred to as complete cell culture medium and standard growth conditions, respectively). Cells were subcultured at approximately 80–90% confluency once a week using 0.5% Trypsin–EDTA (Sigma-Aldrich).

### Formation of reactive oxygen species (ROS)

The formation of ROS in A549 cell**s** was determined using the 2′,7′-dichlorodihydrofluorescein-diacetate assay (H_2_DCF-DA), as described by Roesslein et al. [34]. For experimental details see Additional file 1.

### Cell viability

To assess mitochondrial activity as a measure of cell viability/cell death in A549 cells Cell Titer96® Aqueous One Solution (Promega) containing 3-(4,5-dimethylthiazol-2-yl)-5-(3-carboxymethoxy phenyl)-2-(4-sulfophenyl)-2H (MTS) as a water-soluble tetrazolium compound was used according to the manufacturer’s protocol. In brief, 1.5 × 10^4^ A549 cells were seeded in 200 µL complete cell culture medium in a 96-well plate and grown over night under standard growth conditions. Thereafter medium was removed and cells were incubated for 3 or 24 h in 200 µL complete cell culture medium containing the respective stimuli (abraded particles from MCA-, CC-pressure-treated wood or untreated wood, or eluates derived thereof as described below). Cadmium sulfate (CdSO_4_) in different concentrations served as positive control, untreated cells as negative control. After appropriate incubation times (3, 24 h) medium was replaced by 120 µL of MTS working solution (composed of 20 µL MTS plus 100 µL of phenol-red-free RPMI-1640 w/o supplements) per well and cells were incubated for 60 min at standard growth conditions. Absorption was detected at 490 nm using an ELx800 microplate reader (BioTEK Instruments).

### Data processing

Blank samples treated exactly the same way but containing no cells were run with every cell-based assay. Values given in the graphs are blank-corrected and subsequently normalized to the untreated sample. The mean of at least three independent experiments (each run with technical triplicates) and the corresponding standard deviations are shown.

### Sample preparation for cytotoxicity analysis

Cytotoxicity was assessed in two different scenarios: (i) Abraded particles released from MCA-, CC-pressure-treated as well as untreated wood were diluted in appropriate media and directly applied to cultured cells. (ii) Eluates from the same abraded particles were used to assess the cytotoxicity of active soluble components contained in and released from the wood. Therefore, 4 mg of abraded wood particles per mL elution medium were incubated for 24 h at 37 °C on a rotating platform. Supernatant was collected after centrifugation at 500*g* for 5 min. Elution medium for ROS detection was Hank’s Balanced Salt Solution (HBSS; for experimental details see Additional file 1). For cell viability assessment and cytokine detection (see Additional file 1) eluates were produced in phenol-red free RPMI (without supplements) which was supplemented after centrifugation with 10% FCS, 2 mM l-glutamine, 50 µg/mL penicillin, 50 µg/mL streptomycin, and 100 µg/mL neomycin. The HBSS supernatant as well as the supplemented RPMI supernatant contain the highest possible amount of released components and were labeled “100% eluate”. Serial 1:2 dilutions were performed in the respective media and concordantly termed “50, 25%, etc. eluates”.

### Determination of Cu content in eluates

The Cu content in eluates of the abraded particles from untreated, CC- and MCA-pressure-treated wood was determined by ICP-MS (Sector Field SF-ICP-MS Element 2 from Thermo Finnigan, detection limit: 0.004 µg/L). Prior to analysis, the specimens were acidified with nitric acid (HNO_3_, 65%, Supra Pure) and hydrogen peroxide (H_2_O_2_, 30%, Supra Pure) and subsequently underwent microwave digestion (MLS 1200 MEGA, Milestone, Leutkirch, Germany). Cu plasma standard solutions (1 g/L) were used for calibration.

Production of cytokines. The release of the pro-inflammatory cytokine TNF-α was assessed in macrophages derived from the monocytic cell line THP-1 (ATCC: TIB-202) using the Ready-SET-Go!® Elisa kit (eBioscience) according to the manufacturer’s protocol. For cell culture conditions and experimental details see Additional file 1.

## Results and discussion

### Wood dust particle size

**Fig. 1:**
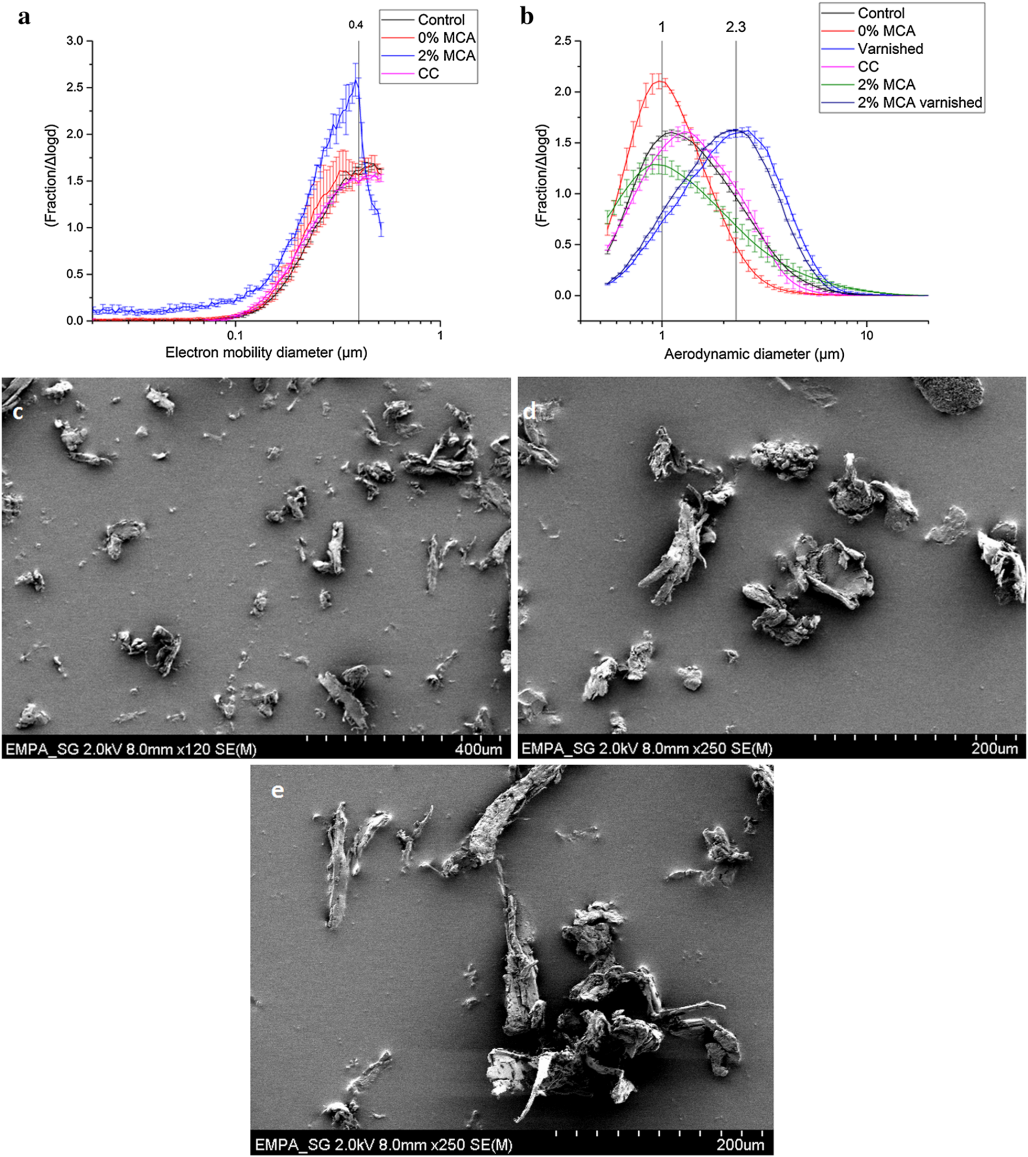
Characterization of the abraded particles. **a** Particle size distributions of untreated wood (control), water-treated wood (0% MCA), MCA-treated wood (2% MCA), and CC-treated wood (CC) measured by SMPS. Most of the abraded particles had a diameter of 400 nm. Data represented as mean of three repetitions. **b** Particle size distributions of untreated wood (control), water-treated wood (0% MCA), varnished wood (varnished), CC-treated wood (CC), MCA-treated wood (2% MCA), and varnished MCA-treated wood (2% MCA varnished) measured by APS. Most of the abraded particles had a diameter of about 1 μm. When varnish is applied, the average diameter shifts towards 2.3 μm. Data represented as mean of three repetitions. **c**, **d** SEM images of wood dust generated by the abrasion process on 2% MCA-treated wood. **e** SEM image of wood dust generated by the abrasion process on untreated wood (control)

The particle size distributions for the different wood samples (untreated, 2% MCA-pressure-treated, 2% CC-pressure-treated, 0% MCA-pressure-treated, varnished, 2% MCA-pressure-treated and varnished) are shown in Fig. 1(a, b). More specific, Fig. 1a represents the particle size distributions measured by SMPS below 1 μm, while Fig. 1b presents the distributions measured by APS above 1 μm. All the samples show a similar pattern below 1 μm, with peaks at about 400 nm; while two different outlines are visible above 1 μm: one for the abraded particles from varnished samples, and another for the abraded particles of unvarnished samples. In the first case the peak is between 700 nm and 1.3 μm, while in the second one it is around 2.3 μm. Therefore, the set up maximizes the release of coarse (PM10), fine (PM2.5) and ultrafine particles (generally defined as smaller than 100 nm). These three particle size fractions are commonly associated with adverse health effects in humans, as demonstrated by Schwartz et al. [35], Raaschou-Nielsen et al. [36], and Oberdörster et al. [37]. In addition, the setting fitted the purpose of detecting any variation in the generated wood dust at the nanoscale, which may have occurred due to the presence of Cu carbonate NPs. In any case, no additional release of a nanosized fraction was observed for the 2% MCA-treated wood.

We could observe how the application of varnishes influences the particle size released increasing the average dimensions, reducing the exposure to ultrafine particles.

The APS results on the aerodynamic particle diameter are in good agreement with the study from Thorpe and Brown [38], in which the wood dust size distribution after different sanding processes was assessed. The mean particle diameter was comprised between 1.52 and 2.65 μm. However, further different abrasive processes, e.g. cutting, grinding, welding, may cause the release of wood dust with different particle size distributions. Despite that, as our abrasive set up maximizes the release of coarse, fine and ultrafine particles, we can suppose that different abrasive processes would not release more nanoparticles than our system.

Our tests focused on Scots pine only, however different wood species may release particles that differ in the size distribution, due to the wood properties, as demonstrated by Lehmann and Fröhlich [39] and Ratnasingam et al. [40]. In the case of MCA-treated wood, the wood species features may also influence the amount of Cu carbonate particles present in the wood after impregnation.

In terms of human exposure, our results indicate that a fraction of the abraded particles produced by the different wood samples could penetrate the lower airways (tracheo-bronchiolar regions or even the alveolar sacs), due to their small size. The application of varnishes alter the size distribution of the abraded particles and by that would shift the particle deposition to the nasopharyngeal and tracheo-bronchiolar regions [41]. However, the broad size range of the particles does not allow a precise quantification of particle deposition in the respiratory tract.

### Wood dust particle morphology

The generated particles from untreated, CC-, and MCA-pressure-treated wood were morphologically assessed by SEM (Fig. 1c–e). Visual inspection of all the SEM images collected confirmed the presence of particles below 10 μm, as well as the presence of bigger particles (10^2^ μm), beyond the APS and SMPS detection limits adopted. In addition, no difference between the different wood samples (Fig. 1d, e) was encountered, indicating no mechanical alteration due to the wood treatments, in accordance with the APS and SMPS results. In all cases, the generated particles appeared mostly fibrous, although irregular and heterogeneous in shape and size. The surfaces were not always flat.

Various studies reported similar features from SEM investigations on wood dust from various wood species [42, 43]. In particular, Mazzoli and Favoni [44] reported no difference in wood dust particle size and morphology from different wood species, suggesting no dissimilarity for in vitro cytotoxicity. However, wood species that are documented to be carcinogenic, e.g. beech [45], were not assessed. In that case, different structures responsible for the increased adverse effects may be observed. In addition, the abrasive process may also generate wood dust particles that differ in size and morphology.

### Cu content in wood dust

By means of ICP-OES and ICP-MS analyses we could assess the different concentrations of Cu in wood dust from untreated and MCA-pressure-treated wood samples, as shown in Table 1. Combining the ICP-OES and ICP-MS results, which are concordant, we determined a baseline amount of Cu in untreated wood at 0.01 ± 0.02 mg/g. Similarly, when the wood was varnished the baseline amount was found at 0.02 ± 0.01 mg/g. When MCA-pressure-treated wood was abraded, the amount of Cu released was 2.02 ± 0.09 mg/g, corresponding to 0.20% w/w of the total amount of treated wood, and it drastically reduced when varnish was applied (0.23 ± 0.01 mg/g). This difference may be due to the higher release of varnish instead of wood, therefore implying that varnishes may prevent release of Cu during mechanical abrasion of treated wood. The amount of Cu release was almost double in CC-pressure-treated wood (4.26 ± 0.01 mg/g). This is due to differences in the formulations: in fact, the amount of Cu in the initial CC formulation doubles the amount in MCA. Since 2% is an economically feasible concentration, generally used in the timber industry, the result indicate that at similar dilutions (2%) MCA-pressure-treated wood would release less Cu due to mechanical abrasion. The percentage of Cu released from MCA-pressure-treated wood is in good agreement with studies on indoor sawing of CCA-treated wood: Decker et al. [46] reported 0.3% Cu in wood dust, while Nygren et al. [47] 0.1%. In addition, a comparison can be made between our results and the ones from the less invasive wiping experiment reported in the EPA report [24]. In fact, in the latter, the amount of Cu released from MCA-pressure-treated wood was lower and comprised between 0.0135 and 0.072 mg.

**Table 1:** Cu content in sawdust particles and eluates thereof

Wood treatment	µg Cu/mg abraded particles	µg Cu/mL medium (eluates^a^) [release in %]
Untreated	0.01 ± 0.02	0.01 ± 0.01
MCA-pressure treated	2.02 ± 0.09	0.36 ± 0.01 [4.4%]
CC-pressure treated	4.26 ± 0.01	0.75 ± 0.01 [4.4%]
RPMI medium (w/o wood)	na	0.00 ± 0.01

^a^4 mg abraded particles were incubated in 1 mL phenolred free RPMI for 24 h at 37 °C on a rotating platform; after centrifugation at 500 g for 5 min supernatants were further processed for ICP-MS measurements

The amount of Cu detected in the wood dust nanosized fraction was below the Cu concentration in the whole wood dust, both from untreated and MCA-pressure-treated wood. In particular the concentration of Cu in the nanosized dust generated by MCA-pressure-treated wood was 1.50 ± 0.30 mg/g (0.15% w/w). Therefore, combining these data with the SMPS results we can conclude that most of the Cu released was bound to the larger wood particles, however a small amount of Cu bound to the nanosized fraction would deposit in the deep lungs, if inhaled. Therefore, toxicological studies are required to fully assess the hazard on human health.

### Cytotoxicity assessment

The most critical exposure route for sawdust particles is the lung. Therefore, we focused our in vitro study on the lung epithelial cell line A549 and macrophages differentiated from the monocytic cell line THP-1. Both cell types are likely to be among the first cell types getting in touch with inhaled particles. We investigated potential adverse effects of sawdust particles abraded from untreated wood, MCA-pressure treated wood and CC-pressure treated wood. Furthermore, to assess the effects caused by soluble compounds, rather than by wood dust per se, eluates from these three types of wood particles were included in the cytotoxicity evaluation. These results were compared to the toxicity induced by direct treatment of lung epithelial cells with MCA and its active components tebuconazole and Cu^2+^ ions from copper sulfate pentahydrate (CuSO_4_·5H_2_O).

**Fig. 2:**
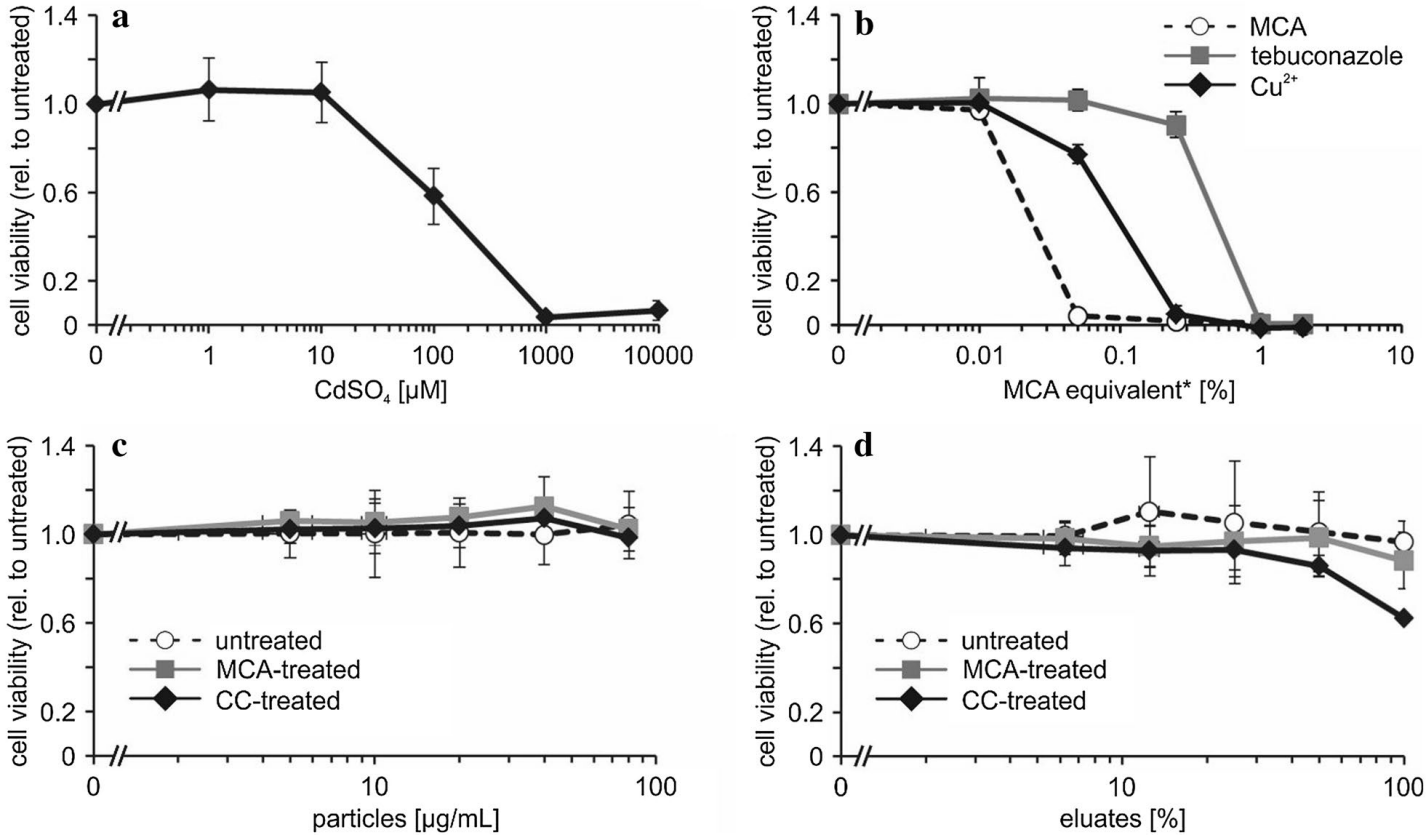
Cell viability assessment in A549 lung epithelial cells. Cells were treated for 24 h with the indicated concentrations of **a** CdSO_4_ as the positive control **b** MCA, tebuconazole and Cu **c** abraded sawdust particles from untreated, MCA-pressure treated and CC-pressure treated wood **d** eluates of the respective wood particles. Cell viability was assessed using the MTS assay. *Tebuconazole and Cu^2+^ were applied in the respective amounts present in MCA as described in Additional file 1

According to the ROS paradigm [34] the interaction of (nano) particles with cells is likely to induce elevated cellular levels of ROS. Subsequent oxidative stress reactions can then cause severe damage to biomolecules (proteins, lipids and nucleic acids), induce inflammatory reactions and finally lead to cell death. Therefore we initially assessed the overproduction of ROS using the DCF assay. As shown in Additional file 1: Figure S1, only the positive controls Sin-1 and MWCNT led to a considerable increase of ROS levels in A549 cells. All eluates and abrasion particles tested did not elevate ROS formation. However, cell death can also be triggered by ROS independent pathways. We therefore investigated cell viability of A549 lung epithelial cells using the MTS assay. The assay internal positive control CdSO_4_ induces cell death in a dose-dependent manner (Fig. 2a) thus indicating that toxicity can be reliably detected under the experimental conditions.

The cytotoxicity of MCA itself was determined up to a concentration of 2% (v/v) in cell culture medium. In parallel, its active compounds tebuconazole and Cu^2+^ were analyzed in equivalent amounts (Fig. 2b; Additional file 1). Our results reveal a toxicity ranking of tebuconazole < Cu^2+^ < MCA, which indicates an additive effect of tebuconazole and Cu^2+^. Further, our results suggest that the cytotoxicity of MCA is likely to be caused by Cu^2+^ ions than nanoparticles.

The highest, technically feasible, concentration of abraded particles that could be applied to A549 cells was 80 µg/mL equaling to a growth area of 47 µg/cm^2^. For all three types of sawdust particles no cytotoxicity could be detected up to this concentration and over an incubation period of 24 h (Fig. 2c). According to Table 1 the highest amount of 80 µg particles from MCA- or CC-pressure-treated wood contain 0.16 or 0.34 µg Cu^2+^, respectively. Measurements of eluates of the respective abraded particles revealed that only a fraction of 4.4% of Cu^2+^ is released into the medium over a period of 24 h (Table 1). Therefore we do not expect concentrations above 0.007 µg/mL or 0.015 µg/mL Cu^2+^ for the two samples, respectively. In relation to Fig. 2b, were Cu^2+^ ion cytotoxicity starts above 5 µg/mL (=0.01%), these values appear very low. However, the following considerations will relate the chosen in vitro doses to an inhalation scenario for wood workers. If we consider an inhalation volume of 1.9 L per breath and roughly 26 breathes per min during heavy exercise [48] we can assume a total volume of 24 m^3^ air to be inhaled during an 8 h working day. According to Decker et al. [46], wood dust concentrations in air may range from 0.6 mg/m^3^ (sampled at outdoor working sites over a period of 229 min) to a maximum of 49 mg/m^3^ (sampled during indoor sanding operations over a period of 127 min). With these data a total amount of 3.8–555 mg inhaled particles per working day can be estimated. Considering 102 m^2^ of total lung surface area [49] and assuming all the wood dust particles to be deposited in the lung we can estimate a total deposited amount of wood dust particles of 0.004–0.545 µg/cm^2^. In this scenario the 47 µg/cm^2^ in vitro dose is a rather high concentration mimicking a repeated exposure over at least 17 weeks (indoor) to a whole lifetime (49 working years; outdoor). Nevertheless, spatially restricted effects due to particle deposition, cellular uptake of particles and potential intracellular Cu^2+^ release cannot be addressed, neither by in vitro toxicity tests nor by the above demonstrated exposure calculations. In summary the doses chosen in the present study adequately reflect a worst case exposure scenario for wood workers.

Furthermore, we analyzed eluates produced from the three types of abraded wood particles and assessed the cytotoxicity of soluble factors released from the sawdust on A549 cells. As shown in Fig. 2d no cytotoxicity could be detected after 24 h of incubation with eluates from untreated as well as MCA-pressure-treated wood. Eluates from CC-pressure-treated wood particles reduced cell viability at the highest concentration tested to 63% viable cells compared to untreated control cultures. This highest eluate concentration (Table 1) contained only 0.8 µg/mL Cu^2+^. As Cu^2+^ ion cytotoxicity started at concentrations beyond 5 µg/mL (=0.01%) (Fig. 2b) Cu^2+^ is most likely not the main reason for the observed effect, but rather chromium. Further investigations are necessary to prove a real human hazard from CC-pressure-treated wood, which was not the scope of the present study. Besides that, our results clearly indicate that there is no additional nano-specific effect, as abraded particles from MCA-pressure-treated wood as well as eluates thereof did not induce any cytotoxicity under the experimental conditions tested. This provides further evidence to the hypothesis that Cu^2+^ ions rather than nanoparticles are responsible for any adverse effects.

Besides cell viability, inflammatory reactions at sublethal concentrations can be an indication for non-acute but nevertheless relevant adverse effects. Therefore we assessed the release of the pro-inflammatory cytokine TNF-α from immune responsive cells in vitro using the enzyme-linked immunosorbent assay (ELISA) technique. We used macrophages differentiated from THP-1 monocytes as the model cell line. Initially, cell viability was investigated to assure sublethal concentrations were applied for subsequent cytokine release experiments. THP-1 macrophages were exposed to the respective stimuli for 8 h and cell viability was assessed using the MTS assay. For technical details see Additional file 1. CdSO_4_ served again as the assay internal positive control and induced cytotoxicity in a dose-dependent manner (Additional file 1: Figure S2a). Following the same experimental design as described for A549 cells MCA and its active components tebuconazole and Cu^2+^ were applied in equivalent amounts (Additional file 1: Figure S2b). In this case, the effects of Cu^2+^ and MCA were comparable, therefore even in this case the effects from MCA appear to be caused by Cu^2+^ ions rather than nanoparticles. Cell viability was affected at concentrations above 0.05% MCA in a dose-dependent manner. All three abraded wood particle types (up to 80 µg/mL) as well as eluates thereof did not induce an adverse response (Additional file 1: Figure S2c, d) in THP-1 macrophages. Accordingly, for cytokine release measurements MCA, tebuconazole and Cu^2+^ were used at concentrations below 0.05% MCA-equivalents and abraded wood particles were used up to 80 µg/mL. Lower eluate concentrations (6.25 to 25.00%) showed an increase in cell viability rather than a decrease. Therefore we used concentrations below 25.00% for ELISA experiments. Treatment with the positive control lipopolysaccharides (LPS) led to a 16- and 25-fold increase in TNF-α release at 10 and 100 ng/mL LPS, respectively (Additional file 1: Figure S3). However, no significant release of TNF-α could be observed after treatment with MCA, its active components, abraded wood particles or eluates thereof at any of the concentrations tested (Additional file 1: Figure S3). Thus, even in this case no specific nano effect was observed.

In summary our findings on the cytotoxicity reveal (1) a toxicity ranking of tebuconazole < Cu^2+^ < MCA (2) no induction of cytotoxicity for abraded particles up to 80 µg/mL (3) only a minor toxicity was found for the highest concentration of eluates resulting from CC-pressure-treated wood, which was only observed for A549 lung epithelial cells, and it is likely due to the presence of chromium in the formulation; most importantly (4) no additional nano hazard (caused by the presence of Cu-based NPs per se) was identified. Furthermore, our cytotoxicity study indicates low adverse effects for low-frequency consumer exposure. However, woodworkers can be continuously exposed to wood dust, in particular since dust-exposed woodworkers do not always wear appropriate respirators approved for wood dust [50]. The wood being processed may have been pressure-treated with Cu-based formulations, and the particles released can increase the adverse effects due to the presence of Cu. However, MCA is likely to be the safest alternative: no nano hazard was evidenced, and the amount of Cu, especially easily bioavailable Cu, in CC was double the amount in MCA. Furthermore, both types of human cells tested showed lower adverse effects (higher cell viability) when compared to cells exposed to CC. In conclusion, the abrasion of MCA-pressure-treated wood does not constitute a nano-specific risk. Nonetheless, further more advanced toxicity studies on tissues and in vivo are required.

## Additional file

**Additional file 1: Figures S1, S2, S3.** Concentration considerations of MCA components; Formation of reactive oxygen species (ROS); Culture conditions and cell viability assessment of THP-1 cells; Production of cytokines.

## References

[1] Jambeck JR, Townsend TG, Solo-Gabriele HM (2008). Landfill disposal of CCA-treated wood with construction and demolition (C&D) debris: arsenic, chromium, and copper concentrations in leachate. Environ Sci Technol.

[2] Townsend T, Solo-Gabriele H, Tolaymat T, Stook K, Hosein N (2003). Chromium, copper, and arsenic concentrations in soil underneath CCA-treated wood structures. Soil Sediment Contam..

[3] Osmose. Consumer safety and product performance of micronized copper technology confirmed. Osmose press release. 2009. http://www.prnewswire.com/news-releases/consumer-safety-and-product-performance-of-micronized-copper-technology-confirmed-65722682.html.

[4] Freeman BMH, Mcintyre CR (2008). A Comprehensive review of copper-based wood preservatives with a focus on new micronized or dispersed copper systems. For Prod J..

[5] Blinova I, Ivask A, Heinlaan M, Mortimer M, Kahru A (2010). Ecotoxicity of nanoparticles of CuO and ZnO in natural water. Environ Pollut.

[6] Griffitt RJ, Weil R, Hyndman K, Denslow N, Powers K, Taylor D, Barber DS (2007). Exposure to copper nanoparticles causes gill injury and acute lethality in zebrafish (Danio rerio). Environ Sci Technol.

[7] Heinlaan M, Kahru A, Kasemets K, Arbeille B, Prensier G, Dubourguier H-C (2011). Changes in the *Daphnia magna* midgut upon ingestion of copper oxide nanoparticles: a transmission electron microscopy study. Water Res.

[8] Heinlaan M, Ivask A, Blinova I, Dubourguier H-C, Kahru A (2008). Toxicity of nanosized and bulk ZnO, CuO and TiO2 to bacteria Vibrio fischeri and crustaceans *Daphnia magna* and Thamnocephalus platyurus. Chemosphere.

[9] Shaw BJ, Al-Bairuty G, Handy RD (2012). Effects of waterborne copper nanoparticles and copper sulphate on rainbow trout (Oncorhynchus mykiss): physiology and accumulation. Aquat Toxicol.

[10] Shi J, Abid AD, Kennedy IM, Hristovaa KR, Silk WK (2011). To duckweeds (*Landoltia punctata*), nanoparticulate copper oxide is more inhibitory than the soluble copper in the bulk solution. Environ Pollut.

[11] Atha DH, Wang H, Petersen EJ, Cleveland D, Holbrook RD, Jaruga P, Dizdaroglu M, Xing B, Nelson BC (2012). Copper oxide nanoparticle mediated DNA damage in terrestrial plant models. Environ Sci Technol.

[12] Bondarenko O, Juganson K, Ivask A, Kasemets K, Mortimer M, Kahru A (2013). Toxicity of Ag, CuO and ZnO nanoparticles to selected environmentally relevant test organisms and mammalian cells in vitro: a critical review. Arch Toxicol.

[13] Chen Z, Meng H, Xing G, Chen C, Zhao Y, Jia G, Wang T, Yuan H, Ye C, Zhao F (2006). Acute toxicological effects of copper nanoparticles in vivo. Toxicol Lett.

[14] Lei R, Wu C, Yang B, Ma H, Shi C, Wang Q, Wang Q, Yuan Y, Liao M (2008). Integrated metabolomic analysis of the nano-sized copper particle-induced hepatotoxicity and nephrotoxicity in rats: a rapid in vivo screening method for nanotoxicity. Toxicol Appl Pharmacol.

[15] Manna P, Ghosh M, Ghosh J, Das J, Sil PC (2012). Liver dysfunction and cellular damage: role of IκBα/NF-κB, MAPKs and mitochondrial signal. Nanotoxicology..

[16] Sarkar A, Das J, Manna P, Sil PC (2011). Nano-copper induces oxidative stress and apoptosis in kidney via both extrinsic and intrinsic pathways. Toxicology.

[17] Meng H, Chen Z, Xing G, Yuan H, Chen C, Zhao F, Zhang C, Zhao Y (2007). Ultrahigh reactivity provokes nanotoxicity: explanation of oral toxicity of nano-copper particles. Toxicol Lett.

[18] Huang Y-W, Wu C, Aronstam RS (2010). Toxicity of transition metal oxide nanoparticles: recent insights from in vitro studies. Materials..

[19] Karlsson HL, Cronholm P, Gustafsson J, Möller L (2008). Copper oxide nanoparticles are highly toxic: a comparison between metal oxide nanoparticles and carbon nanotubes. Chem Res Toxicol.

[20] Karlsson HL, Holgersson A, Möller L (2008). Mechanisms related to the genotoxicity of particles in the subway and from other sources. Chem Res Toxicol.

[21] Karlsson HL, Gustafsson J, Cronholm P, Möller L (2009). Size-dependent toxicity of metal oxide particles—a comparison between nano- and micrometer size. Toxicol Lett.

[22] Lanone S, Rogerieux F, Geys J, Dupont A, Maillot-Marechal E, Boczkowski J, Lacroix G, Hoet P (2009). Comparative toxicity of 24 manufactured nanoparticles in human alveolar epithelial and macrophage cell lines. Part Fibre Toxicol..

[23] Midander K, Cronholm P, Karlsson HL, Elihn K, Möller L, Leygraf C, Wallinder IO (2009). Surface characteristics, copper release, and toxicity of nano- and micrometer-sized copper and copper(II) oxide particles: a cross-disciplinary study. Small.

[24] US Environmental Protection Agency. Release of micronized copper particles from pressure-treated wood. 600/R-14/365. 2014. http://www.epa.gov/ord.

[25] Stirling R, Ruddick JNR, Morris PI (2015). Characterization of copper in leachates from ACQ- and MCQ-treated wood and its effect on basidiospore germination. Wood Fiber Sci.

[26] Stirling R, Morris PI (2010). Mobility of copper from MCQ in shell-treated wood exposed above ground. IRG/WP 10-30534.

[27] Kartal SN, Green F, Clausen CA (2009). Do the unique properties of nanometals affect leachability or efficacy against fungi and termites?. Int Biodeterior Biodegrad.

[28] Wang L, Kamdem P. Copper leached from micronized copper quaternary (MCQ) treated wood: influence of the amount of copper in the formulations. In: proceedings of the 55th international convention of society of wood science and technology, 27–31 Aug 2012, Beijing; 2012. Paper PS-66. http://www.swst.org/meetings/AM12/pdfs/papers/PS-66.pdf. Accessed Aug 2016.

[29] Platten WE, Sylvest N, Warren C, Arambewela M, Harmon S, Bradham K, Rogers K, Thomas T, Luxton TP (2016). Estimating dermal transfer of copper particles from the surfaces of pressure-treated lumber and implications for exposure. Sci Total Environ.

[30] Santiago-Rodríguez L, Griggs JL, Bradham KD, Nelson C, Luxton T, Platten WE, Rogers KR (2015). Assessment of the bioaccessibility of micronized copper wood in synthetic stomach fluid. Environ Nanotechnol Monit Manag..

[31] Civardi C, Schubert M, Fey A, Wick P, Schwarze FWMR (2015). Micronized copper wood preservatives: efficacy of ion, nano, and bulk copper against the brown rot fungus Rhodonia placenta. PLoS ONE.

[32] European Committee for Standardization. ENV 807. (2001). Wood preservatives-determination of the effectiveness against soft rotting micro-fungi and other soil inhabiting micro-organisms.

[33] Schlagenhauf L, Chu BTT, Buha J, Nüesch F, Wang J (2012). Release of carbon nanotubes from an epoxy-based nanocomposite during an abrasion process. Environ Sci Technol.

[34] Roesslein M, Hirsch C, Kaiser JP, Krug HF, Wick P (2013). Comparability of in vitro tests for bioactive nanoparticles: a common assay to detect reactive oxygen species as an example. Int J Mol Sci.

[35] Schwartz J, Dockery DW, Neas LM (1996). Is daily mortality associated specifically with fine particles?. J Air Waste Manag Assoc.

[36] Raaschou-Nielsen O, Andersen ZJ, Beelen R, Samoli E, Stafoggia M, Weinmayr G, Hoffmann B, Fischer P, Nieuwenhuijsen MJ, Brunekreef B (2013). Air pollution and lung cancer incidence in 17 European cohorts: prospective analyses from the European study of cohorts for air pollution effects (ESCAPE). Lancet Oncol..

[37] Oberdörster G, Celein RM, Ferin J, Weiss B (1995). Association of particulate air pollution and acute mortality: involvement of ultrafine particles?. Inhal Toxicol..

[38] Thorpe A, Brown RC (1994). Measurements of the effectiveness of dust extraction systems of hand sanders used on wood. Ann Occup Hyg.

[39] Lehmann E, Fröhlich N (1988). Particle size distribution of wood dust at the workplace. J Aerosol Sci.

[40] Ratnasingam J, Scholz F, Natthondan V, Graham M (2011). Dust-generation characteristics of hardwoods during sanding processes. Eur J Wood Wood Prod..

[41] Oberdörster G, Oberdörster E, Oberdörster J (2005). Nanotoxicology: an emerging discipline evolving from studies of ultrafine particles. Environ Health Perspect.

[42] Atuanya CU, Ibhadode AOA (2011). Characterization of Okhuen (*Brachystegia nigerica*) wood as a potential reinforcement for polymer composites. Int J Eng Technol..

[43] Gómez Yepes ME, Cremades LV (2011). Characterization of wood dust from furniture by scanning electron microscopy and energy-dispersive X-ray analysis. Ind Health.

[44] Mazzoli A, Favoni O (2012). Particle size, size distribution and morphological evaluation of airborne dust particles of diverse woods by scanning electron microscopy and image processing program. Powder Technol.

[45] Klein RG, Schmezer P, Amelung F, Schroeder HG, Woeste W, Wolf J (2001). Carcinogenicity assays of wood dust and wood additives in rats exposed by long-term inhalation. Int Arch Occup Environ Health.

[46] Decker P, Cohen B, Butala JH, Gordon T (2002). Exposure to wood dust and heavy metals in worker using CCA pressure-treated wood. AIHA J.

[47] Nygren O, Nilsson CA, Lindahl R (1992). Occupational exposure to chromium, copper and arsenic during work with impregnated wood in joinery shops. Ann Occup Hyg.

[48] Gangwa S, Brown JS, Wang A, Houck KA, Dix DJ, Kavlock RJ, Cohen Hubal EA (2011). Informing selection of nanomaterial concentrations for ToxCast in vitro testing based on occupational exposure potential. Environ Health Perspect.

[49] Sargent LM, Porter DW, Staska LM, Hubbs AF, Lowry DT, Battelli L, Siegrist KJ, Kashon ML, Mercer RR, Bauer AK (2014). Promotion of lung adenocarcinoma following inhalation exposure to multi-walled carbon nanotubes. Part Fibre Toxicol..

[50] Alwis U, Mandryk J, Hocking AD, Lee J, Mayhew T, Baker W (1999). Dust exposures in the wood processing industry. Am Ind Hyg Assoc J.

