# Supplementary material for: Release of copper-amended particles from micronized copper-pressure-treated wood during mechanical abrasion
Source: J Nanobiotechnology. 2016 Nov 28;14:77. doi: 10.1186/s12951-016-0232-7 (PMC5126862; doi:10.1186/s12951-016-0232-7)
Supplement: Supplementary file 1 — Additional file 1: Figures S1, S2, S3. Concentration considerations of MCA components; Formation of reactive oxygen species (ROS); Culture conditions and cell viability assessment of THP-1 cells; Production of cytokines. [file 12951_2016_232_MOESM1_ESM.docx]

Release of Copper- Amended Particles from Micronized Copper-Pressure-Treated Wood during Mechanical Abrasion

Chiara Civardi^1,2‡^*, Lukas Schlagenhauf^3,4,5‡^, Jean-Pierre Kaiser^6^, Cordula Hirsch^6^, Claudio Mucchino^7^, Adrian Wichser^4,6^, Peter Wick^6^, Francis W.M.R. Schwarze^1^*.

**Concentration considerations of MCA components**

The active components of MCA are tebuconazole and Cu-carbonate (CuCO_3_Cu(OH)_2_) in its nanoparticulate form. MCA itself is commonly applied to wood as a 2% solution in water. For cell viability testing we assumed a worst case scenario with human exposures at a maximum of 2%. Tebuconazole as well as Cu concentrations were calculated accordingly to match the respective amount present in e.g. 2% MCA. CuSO_4_∙5H_2_O was used as a soluble Cu^2+^ source.

**Formation of reactive oxygen species (ROS)**

A549 cells were seeded into 96-well plates at a density of 2 x 10^4^ cells per well in a volume of 200 µL complete cell culture medium one day prior to treatment. Cells were incubated with 50 µM H_2_DCF-DA (Molecular Probes) in HBSS at standard growth conditions. After two washing steps with pre-warmed HBSS cells were treated with abraded wood particles (MCA-, CC-pressure-treated and untreated) and eluates thereof. The nitrite oxide donor 3-morpholinosydnonimine hydrochloride (Sin-1; 50 µM) as well as multi-walled carbon nanotubes (MWCNT; Baytubes, Bayer Technologies; 10 µg/mL and 20 µg/mL) were used as positive controls. Fluorescence intensity was measured after 1, 2, 3 and 4 h from the same plate using a Mithras2 plate reader (Berthold Technologies) at an excitation wavelength of 485 nm and an emission wavelength of 528 nm. Only values from the 3 h measurement are shown. The mean of at least three independent experiments (each run with technical triplicates) and the corresponding standard deviations are shown.

| 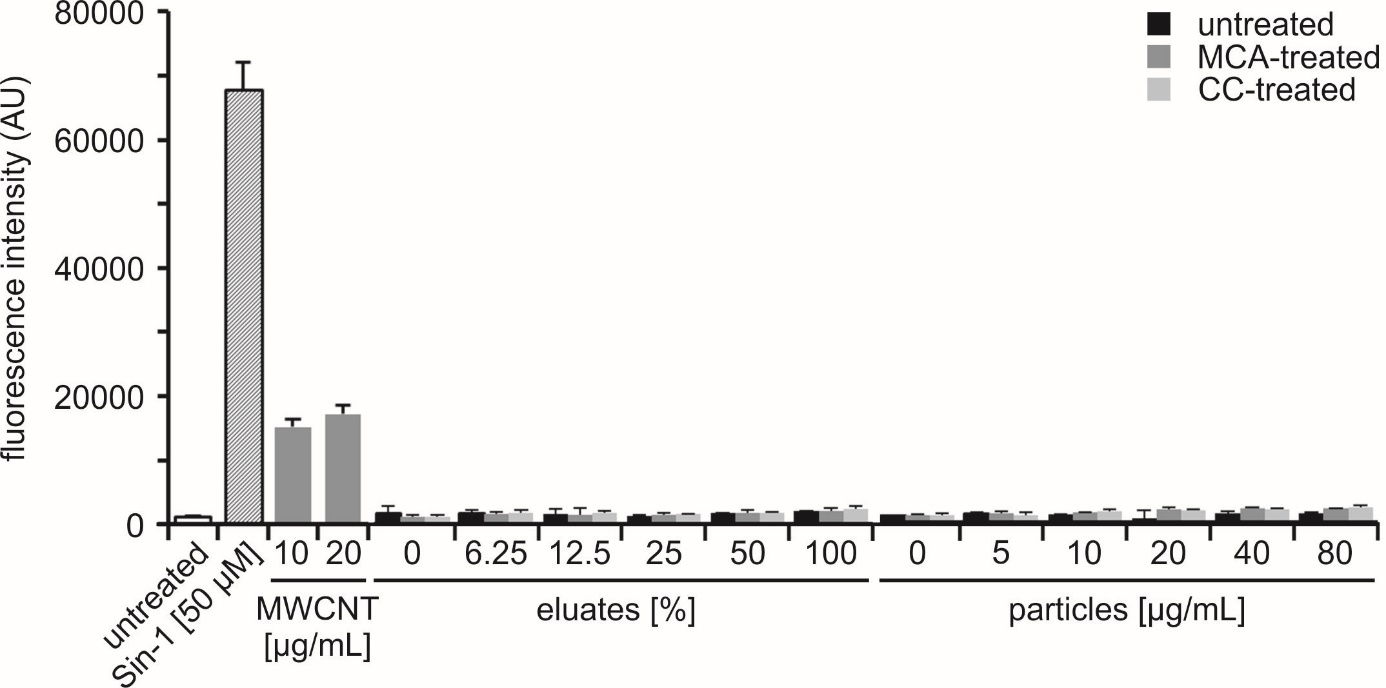 |
| --- |
| **Figure S1.** None of the particles and eluates tested enhanced ROS production in A549 cells. ROS formation was measured in A549 cells after three hours of incubation with indicated concentrations of abraded wood particles as well as eluates thereof. Sin-1 (50 µM) as well as MWCNT served as the positive controls. Sin-1: 3-morpholinosydnonimine hydrochloride. |

**Culture conditions and cell viability assessment of THP-1 cells**

The human monocytic cell line THP-1 (ATCC: TIB-202) was grown as cell suspension in complete cell culture medium under standard growth conditions in complete cell culture medium (RPMI-1640 supplemented with 10% FCS (Lonza), 2 mM L-glutamine (Gibco), 50 µg/mL penicillin (Gibco), 50 µg/mL streptomycin (Gibco), and 100 µg/mL neomycin (Gibco)). Subculturing was performed by replacement of medium when the cell density reached 8 x 10^5^ cells/mL. Cell concentrations were not allowed to exceed 10^6^ cells/mL.

To determine sublethal concentrations of abraded particles, eluates thereof as well as MCA components (MCA itself, tebuconazole, Cu^2+^) cell viability was assessed using the Cell Titer96® AQueous One Solution (Promega) as described in the main text with the following exceptions. 8 x 10^4^ THP-1 monocytes were seeded in 200 µl complete cell culture medium containing 200 nM PMA (12-myristate-13-acetate) per 96-well. Cells were differentiated into adherently growing macrophages in the presence of PMA for 72 h under standard growth conditions. After one washing step with pre-warmed phosphate buffered saline (PBS) cells were treated with the compounds of interest in complete cell culture medium (w/o PMA) for 8 h. Thereafter medium was replaced by 120 µL of MTS working solution, cells were incubated for 60 min at standard growth conditions and absorption was read at 490 nm using an ELx800 microplate reader (BioTEK Instruments).

| **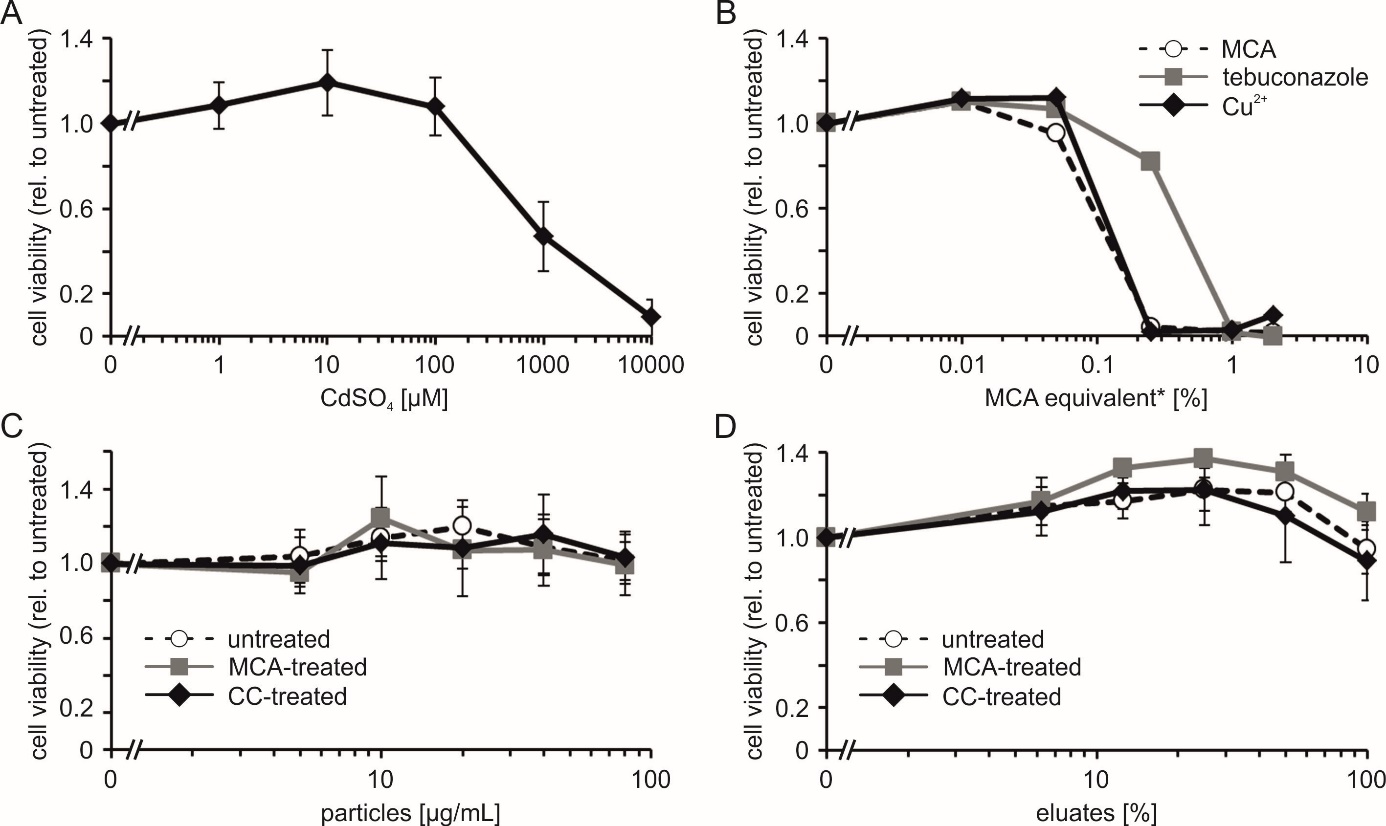** |
| --- |
| **Figure S2.** No acute cytotoxicity was detected after treatment of THP-1 cells with abraded wood particles and eluates thereof. MCA as well as tebuconazole and Cu^2+^ induce a dose dependent toxicity in THP-1 cells. *Tebuconazole and Cu^2+^ were applied in the respective amounts present in MCA as described above. |

**Production of cytokines**

4 x 10^4^ THP-1 monocytes were seeded in 200 µL complete cell culture medium per well of a 96-well plate and were differentiated into macrophages over period of 72 h in the presence of 200 nM as described above. After one washing step with pre-warmed PBS cells were treated with the MCA compounds, abraded particles and eluates thereof for 8 h under standard growth conditions (w/o PMA). Cells treated with 10 ng/mL and 100 ng/mL lipopolysaccharide (LPS) served as positive control samples, untreated cells as negative control samples. Cell-free supernatants were harvested by centrifugation (500g, 5 min) and frozen at -80°C until subsequent cytokine analysis. TNF-α concentrations were determined using the Ready-SET-Go!® Elisa kit (eBioscience) according to the manufacturer’s protocol. Absorbance values were measured at 630 nm using a Mithras2 plate reader (Berthold Technologies) and normalized to the untreated control sample. The mean of three independent experiments (each run with technical duplicates) and the corresponding standard deviations are shown.

| **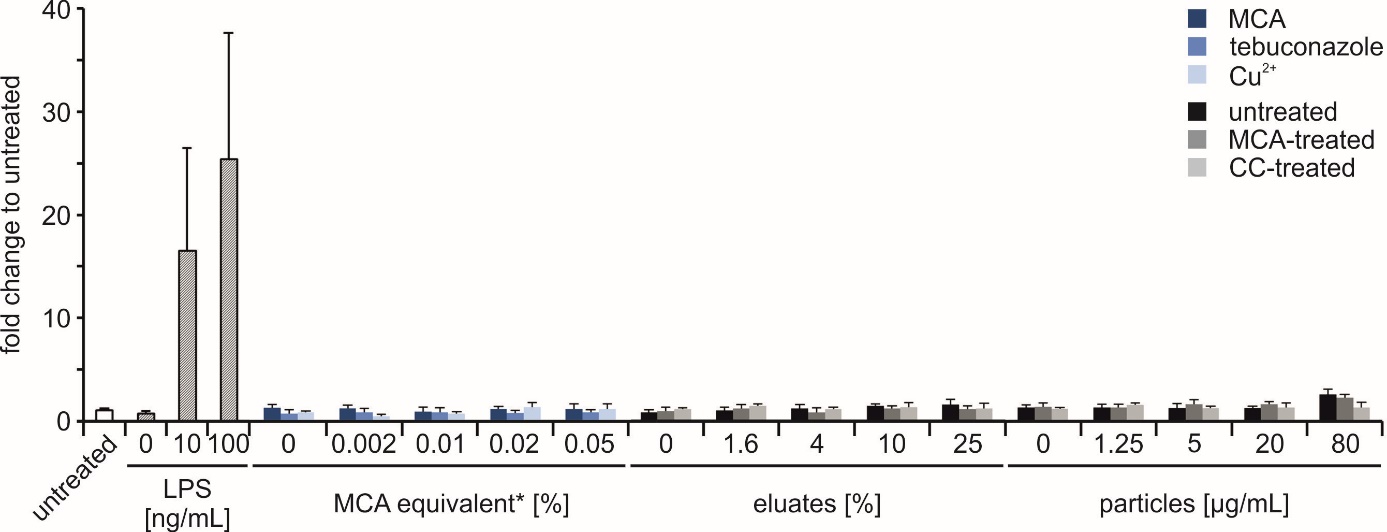** |
| --- |
| **Figure S3.** None of the substances tested induced the release of the pro-inflammatory cytokine TNF-α. *Tebuconazole and Cu^2+^ were applied in the respective amounts present in MCA as described above. |
